# Impact of model-informed precision dosing in adults receiving vancomycin via continuous infusion: a randomized, controlled clinical trial

**DOI:** 10.1186/s13063-024-07965-6

**Published:** 2024-02-16

**Authors:** Glenn Van Wynsberge, Veerle Grootaert, Franky Buyle, Jens Van Praet, Roos Colman, Ine Moors, Annemie Somers, Diana Huis in ‘t Veld, Pieter De Cock

**Affiliations:** 1 Department of Pharmacy, Ghent University Hospital, Ghent, Belgium; 2 Department of Pharmacy, General Hospital Sint-Jan Bruges, Bruges, Belgium; 3 Department of Nephrology and Infectious Diseases, General Hospital Sint-Jan Bruges, Bruges, Belgium; 4 Biostatistics Unit, Faculty of Medicine and Health Sciences, Ghent University, Ghent, Belgium; 5 Department of Hematology, Ghent University Hospital, Ghent, Belgium; 6 Pharmaceutical Care Unit, Faculty of Pharmaceutical Sciences, Ghent University, Ghent, Belgium; 7 Department of General Internal Medicine and Infectious Diseases, Ghent University Hospital, Ghent, Belgium; 8 Department of Basic and Applied Medical Sciences, Faculty of Medicine and Health Sciences, Ghent University, Ghent, Belgium; 9 Department of Hematology, General Hospital Sint-Jan Bruges, Bruges, Belgium; 10 Department of Orthopedics, Ghent University Hospital, Ghent, Belgium; 11 Department of Orthopedics, General Hospital Sint-Jan Bruges, Bruges, Belgium

**Keywords:** Antibiotic, Vancomycin, Therapeutic drug monitoring, Model-informed precision dosing, Continuous infusion, Randomized controlled trial

## Abstract

**Background:**

Vancomycin is a commonly prescribed antibiotic to treat gram-positive infections. The efficacy of vancomycin is known to be directly related to the pharmacokinetic/pharmacodynamic (PK/PD) index of the area under the concentration-time curve (AUC) divided by the minimal inhibitory concentration (MIC) of the pathogen. However, in most countries, steady-state plasma concentrations are used as a surrogate parameter of target AUC/MIC, but this practice has some drawbacks. Hence, direct AUC-guided monitoring of vancomycin using model-informed precision dosing (MIPD) tools has been proposed for earlier attainment of target concentrations and reducing vancomycin-related nephrotoxicity. However, solid scientific evidence for these benefits in clinical practice is still lacking. This randomized controlled trial (RCT) aims to investigate the clinical utility of MIPD dosing of vancomycin administered via continuous infusion in hospitalized adults.

**Methods:**

Participants from 11 wards at two Belgian hospitals are randomly allocated to the intervention group or the standard-of-care comparator group. In the intervention group, clinical pharmacists perform dose calculations using CE-labeled MIPD software and target an AUC24h of 400 to 600 mg × h/L, whereas patients in the comparator group receive standard-of-care dosing and monitoring according to the institutional guidelines. The primary endpoint is the proportion of patients reaching the target AUC24h/MIC of 400–600 between 48 and 72 h after start of vancomycin treatment. Secondary endpoints are the proportion of patients with (worsening) acute kidney injury (AKI) during and until 48 h after stop of vancomycin treatment, the proportion of patients reaching target AUC24h/MIC of 400–600 between 72 and 96 h after start of vancomycin treatment, and the proportion of time within the target AUC24h/MIC of 400–600.

**Discussion:**

This trial will clarify the propagated benefits and provide new insights into how to optimally monitor vancomycin treatment.

**Trial registration:**

EudraCT number: 2021-003670-31. Registered June 28, 2021. ClinicalTrials.gov identifier: NCT05535075. Registered September 10, 2022. Protocol version 3, protocol date: April 21, 2023.

## Administrative information

Note: the numbers in curly brackets in this protocol refer to SPIRIT checklist item numbers. The order of the items has been modified to group similar items (see http://www.equator-network.org/reporting- guidelines/spirit-2013-statement-defining-standard-protocol-items-for-clinical-trials/).

**Table:** 

Title {1}	Impact of model-informed precision dosing in adults receiving vancomycin via continuous infusion: a randomized, controlled clinical trial.
Trial registration {2a and 2b}.	EudraCT number: 2021-003670-31. ClinicalTrials.gov Identifier: NCT05535075.
Protocol version {3}	Protocol version 3. Protocol date: April 21, 2023.
Funding {4}	Vancomycin treatment is part of routine care. The MIPD software is provided in kind by the company Insight RX for conducting the trial.
Author details {5a}	Glenn Van Wynsberge^1^, Veerle Grootaert^2^, Franky Buyle^1^, Jens Van Praet^3^, Roos Colman^4^, Ine Moors^5^, Annemie Somers^1,6^, Diana Huis in ’t Veld^7^, Pieter De Cock^1,8^ ^1^ Department of Pharmacy, Ghent University Hospital, Belgium ^2^ Department of Pharmacy, General Hospital Sint-Jan Bruges, Belgium ^3^ Department of Nephrology and Infectious Diseases, General Hospital Sint-Jan Bruges, Belgium ^4^ Biostatistics Unit, Faculty of Medicine and Health Sciences, Ghent University, Belgium ^5^ Department of Hematology, Ghent University Hospital, Belgium ^6^ Faculty of Pharmaceutical Sciences, Ghent University, Belgium ^7^ Department of General Internal Medicine and Infectious Diseases, Ghent University Hospital, Belgium ^8^ Department of Basic and Applied Medical Sciences, Ghent University Hospital, Belgium
Name and contact information for the trial sponsor {5b}	Ghent University Hospital, Ghent, Belgium Contact: Pieter De Cock. Tel: +3293322969 Email: pieter.decock@uzgent.be.
Role of sponsor {5c}	Responsibilities of the sponsor are: • Central data collection and verification of reportable events. • Notifying investigators and reporting of SUSARs within required timelines. • Preparing standard tables and other relevant information for the Development Safety Update Report (DSUR) in collaboration with the Chief Investigator (CI) and ensuring timely submission to the regular authorities and EC. • Submission of the annual progress reports, including safety summary and deviations.

## Introduction

### Background and rationale {6a}

Vancomycin is a commonly prescribed antibiotic to treat gram-positive infections, predominantly in the empirical and directed treatment of septicemia, endocarditis, bone infections, and skin and soft-tissue infections. Methicillin-resistant *Staphylococcus aureus* (MRSA) is an important causative pathogen of these community or healthcare associated infections, with vancomycin as the cornerstone therapy [1, 2]. The efficacy of vancomycin is known to be directly related to the pharmacokinetic/pharmacodynamic (PK/PD) index of the area under the concentration-time curve (AUC) divided by the minimal inhibitory concentration (MIC) of the pathogen (AUC/MIC). The advocated steady-state AUC24h/MIC for favorable clinical outcome in humans with MRSA and enterococci infections is at least 400 [3]. An AUC24h of 600 mg/L × h or higher is known to be independently associated with an increased risk of developing acute kidney injury (AKI), indicating its narrow therapeutic index [4]. Vancomycin is commonly initiated on a mg per kg basis according to institutional guidelines. After reaching steady-state conditions, approximately 24 h after the start of a continuous infusion dosing regimen, therapeutic drug monitoring (TDM) is performed to ensure attainment of target concentrations [3]. If necessary, predefined dose adjustments are performed, aiming at a steady-state plasma concentration for continuous infusions [5].

Pharmacokinetic (PK) modeling involves the development of mathematical models to describe a drug’s PK behavior. These models are used to predict the drug PK in the individual patient. In the Bayesian approach, estimates of an individual patient’s PK parameters (e.g., clearance and volume of distribution) are provided using the PK model, patient characteristics (e.g., weight, age, serum creatinine) and vancomycin blood concentration measurements. PK parameters can be re-estimated if new sources of information, such as changing kidney function or repeat blood concentration measurements, become available. These PK estimates, referred to as the Bayesian conditional posteriors, can be used to estimate a patient-specific AUC and guide dose optimization. In recent decades, model-informed precision dosing (MIPD) has been used to ensure efficacious antibiotic treatment with significant success [5–7]. For vancomycin, Bayesian AUC-guided MIPD for intermittent dosing regimens has been shown to lead to significantly decreased nephrotoxicity, lower total vancomycin doses, reduced blood sampling, and shorter length of therapy without compromising efficacy, compared to trough-only monitoring [3, 4, 8]. In the latest consensus guideline of the American Society of Health-System Pharmacists, the Infectious Diseases Society of America, the Pediatric Infectious Diseases Society, and the Society of Infectious Diseases Pharmacists, Bayesian AUC-guided vancomycin dosing is recommended for treatment of MRSA infections and is already the standard in some countries [3, 9, 10]. However, solid scientific evidence for the use of MIPD for continuous infusion regimens remains scarce. The overall objective of this study is to investigate the clinical utility of MIPD of vancomycin administered via continuous infusion in hospitalized adults.

### Objectives {7}

#### Primary objectives

This study aims to test the primary hypothesis that AUC/MIC-based vancomycin dosing, using a MIPD calculator, increases the proportion of patients reaching the therapeutic target AUC24h/MIC (400–600) between 48 and 72 h after the start of treatment, compared to the use of standard-of-care continuous infusion dosing regimens with therapeutic drug monitoring.

#### Secondary objectives

This study also aims to test the following secondary hypotheses:

1. AUC/MIC-based vancomycin dosing, using a MIPD calculator, reduces the proportion of patients with (worsening) acute kidney injury during and until 48 h after stopping treatment with vancomycin, compared to the use of standard-of-care continuous infusion dosing regimens with therapeutic drug monitoring;
2. AUC/MIC-based vancomycin dosing, using a MIPD calculator, increases the proportion of patients reaching the therapeutic target AUC24h/MIC (400–600) between 72 and 96 h after the start of treatment, compared to the use of standard-of-care continuous infusion dosing regimens with therapeutic drug monitoring;
3. AUC/MIC-based vancomycin dosing, using a MIPD calculator, increases the proportion of time within the therapeutic target range AUC24h/MIC (400–600) during vancomycin treatment, compared to the use of standard-of-care continuous infusion dosing regimens with therapeutic drug monitoring.

#### Tertiary objectives

This study also aims to test the following tertiary hypotheses:

1. AUC/MIC-based vancomycin dosing, using a MIPD calculator, reduces the number of (additional) blood samples to first target attainment, compared to the use of standard-of-care continuous infusion dosing regimens with therapeutic drug monitoring;
2. AUC/MIC-based vancomycin dosing, using a MIPD calculator, reduces the cumulative number of blood samples during treatment, compared to the use of standard-of-care continuous infusion dosing regimens with therapeutic drug monitoring;
3. AUC/MIC-based vancomycin dosing, using a MIPD calculator, reduces the number of dose adjustments to first target attainment during treatment, compared to the use of standard-of-care continuous infusion dosing regimens with therapeutic drug monitoring;
4. AUC/MIC-based vancomycin dosing, using a MIPD calculator, reduces the cumulative vancomycin dose and corresponding AUC during vancomycin treatment, compared to the use of standard-of-care continuous infusion dosing regimens with therapeutic drug monitoring.

### Trial design {8}

This study is a prospective individually randomized controlled multicenter superiority clinical trial. Patients are randomized to the standard-of-care comparator or intervention arm with an allocation ratio of 1:1.

## Methods: participants, interventions, and outcomes

### Study setting {9}

This trial is currently recruiting patients in 11 wards for non-critically ill adults at two Belgian hospitals: Ghent University Hospital, an academic center, and general hospital Sint-Jan Bruges.

### Eligibility criteria {10}

Patients aged 18 years or older are eligible for inclusion if:

- They are admitted to a participating ward unit (hematology, orthopedic, gastrointestinal surgery or internal medicine);
- They have a suspected or confirmed Gram-positive infection;
- Intravenous continuous infusion vancomycin treatment has started or is planned to be started;
- They or their legal representative signed the informed consent form (ICF);
- They were not previously enrolled in this trial.

Patients are not eligible if:

- Their serum creatinine level at inclusion is above 2.5 mg/dL;
- They are undergoing extracorporeal treatment at inclusion (e.g., extracorporeal membrane oxygenation, dialysis, body cooling);
- The patient’s death is deemed imminent and inevitable.

A patient restarted on vancomycin within the 20-day study period is not considered a new enrollment and should receive the treatment assigned for in the first episode of vancomycin treatment.

### Who will take informed consent? {26a}

Informed consent is obtained by a trained and delegated physician. The physician informs the patient or legal representatives about the study whenever a vancomycin therapy has started and the patient meets the eligibility criteria. The eligible patients receive both an oral explanation of the study and a participant information sheet (PIS) with a participant consent form. Written informed consent of eligible participants is obtained prior to inclusion by discussing the nature, objectives, and possible adverse events associated with their participation in the study. A copy of the information sheet and the signed and dated consent form are supplied to the patient or legal representatives providing written consent.

### Additional consent provisions for collection and use of participant data and biological specimens {26b}

N/A: only data available in the electronic medical record of the patients are collected for the purposes of this trial. No additional consent provisions are needed.

## Interventions

### Explanation for the choice of comparators {6b}

The overall objective of the study is to evaluate the clinical utility of MIPD in adults receiving vancomycin via continuous infusion. Therefore, a proper comparator is the current standard-of-care practice. In this comparator arm, assessment of the vancomycin starting dose and dose adjustments based on TDM concentration are performed according to the institutional dosing regimen and dose adjustment nomograms. Dosing regimens are prescribed by the attending physician. First, a loading dose between 20 and 30 mg per kg total body weight (TBW) is initiated and administered over a period of 2 h. The subsequent maintenance dose is based on the TBW and the patient’s estimated kidney function and administered as a 24-h continuous infusion. The first TDM sample is typically drawn 24 h after start infusion of the loading dose and dose adjustments are performed in accordance to predefined nomograms and TDM concentration target ranges (Ghent University Hospital: 20–25 mg/L for standard infections and 25–30 mg/L for severe infections; general hospital Sint-Jan Bruges: 20–25 mg/L) [3, 11, 12]. If dose adjustment is necessary, repeat TDM samples are typically drawn 24 h after dose adjustment. Otherwise, routine monitoring of TDM concentration and serum creatinine is performed at least twice a week. Clinical pharmacists, as per standard-of-care, monitor compliance with the institutional guidelines daily and provide pharmaceutical care advice with regard to dosing and therapeutic drug monitoring. Interpretation of TDM concentrations is assured within an 8-h interval from the lab report becoming available.

### Intervention description {11a}

**Fig. 1:**
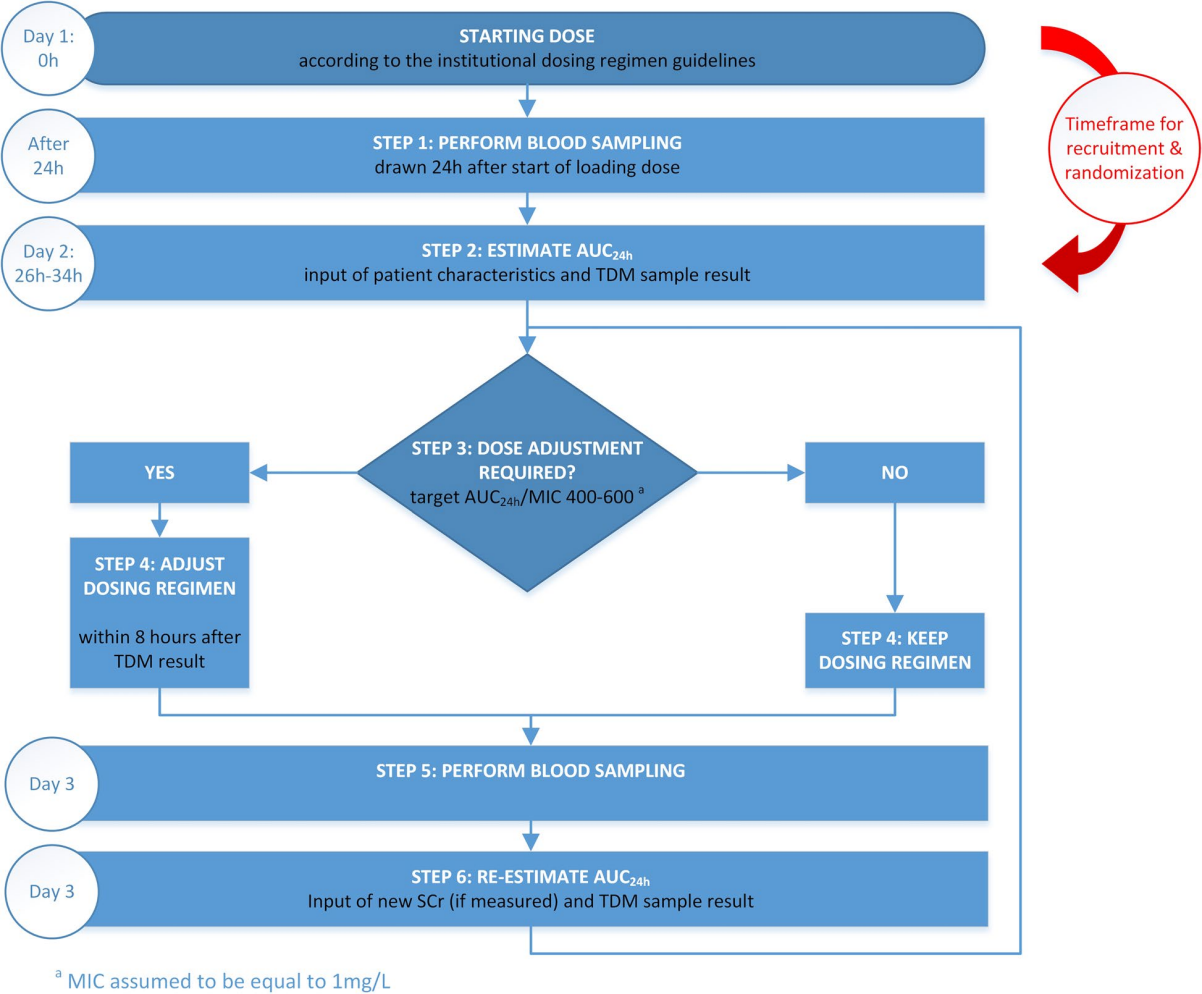
Dosing and blood sampling workflow for patients in the intervention arm

The MIPD software for AUC24h assessment in this study is the InsightRX Nova web application [13]. A target AUC24h/MIC is defined between 400 to 600, assuming an MIC of 1 mg/L. In case of severe infections, typically the higher end of the target range is used. The vancomycin PK model by Colin et al. is used within this MIPD software package. It is a meta-analysis PK model, pooling PK data from premature neonates to elderly, intensive care unit (ICU), and obese patients (*n* = 2554 patients). In this PK model, current weight, age, and serum creatinine are covariates for dose forecasting, eventually in combination with individual TDM concentrations [14]. This model is chosen since it was one of the best predictive PK models in a retrospective fit-for-purpose validation using vancomycin PK data from patients of three Belgian hospitals, including the Ghent University Hospital, and general hospital Sint-Jan Bruges [15]. As for patients in the comparator arm, the vancomycin loading dose and continuous infusion are initiated by the attending physician according to the institutional dosing guidelines. As for the comparator arm first TDM samples are typically drawn 24 h after start infusion of the loading dose (Fig 1). This TDM concentration is registered in the MIPD software together with patient’s most recent TBW, serum creatinine concentrations, and vancomycin dosing details for AUC assessment. If AUC-based dose adjustment is necessary, repeat TDM samples are typically drawn after 24 h. Otherwise, routine monitoring of serum creatinine and TDM concentrations are performed at least twice a week. Every time a TDM concentration becomes available, AUC24h is assessed using Bayesian estimation by trained clinical pharmacists within an 8-h interval. AUC estimations are based on all dosing details, all available TDM concentrations and measurements of TBW and serum creatinine. All dose calculations and eventual dose adjustments are performed and immediately communicated to the attending physician and nurses for implementation. The InsightRX software has a number of real-time checks and warnings implemented, e.g., warnings for duplicate doses or TDM measurements or warnings for out-of-range weight, serum creatinine, or vancomycin blood concentration.

### Criteria for discontinuing or modifying allocated interventions {11b}

The patient or legal representative of the patient may withdraw (written or oral) consent at any time during the study. The patient receives the standard-of-care treatment from the moment of withdrawal of consent. If the patient is not withdrawn from the study but the trial intervention (i.e., the use of the MIPD dosing calculator for dosing vancomycin) is prematurely discontinued for any reason before day 20, the patient receives standard-of-care treatment.

Reasons for premature discontinuation of the use of the MIPD dosing calculator may include the following:

- The patient is requiring extracorporeal treatment (dialysis, extracorporeal membrane oxygenation, body cooling);
- The patient develops acute kidney injury Kidney Disease Improving Global Outcomes (KDIGO) class 3;
- The treating physician’s decision due to other safety reasons than kidney injury;
- The patient is transferred to a ward unit in a hospital not participating in the study;
- The patients’ withdrawal of informed consent.

Note: If a patient is readmitted to one of the participating ward units, the study assigned dosing method needs to be followed within the 20-day study period if still receiving or restarted on vancomycin.

A reason for premature discontinuation of vancomycin administration may be the treating physician’s decision due to safety reasons, e.g., kidney injury. If vancomycin is permanently stopped within a treatment episode according to standard-of-care treatment decisions (e.g., de-escalation of antibiotic treatment, switch of antibiotic since vancomycin is not an appropriate treatment choice for the identified pathogen of the infection), this is not considered a premature discontinuation.

### Strategies to improve adherence to interventions {11c}

All members of the study team are duly trained on Good Clinical Practices, the study protocol, and their distinct responsibilities in this trial, e.g., patient screening, obtaining informed consent, and MIPD dose calculations. Procedures, worksheets, and step-by-step plans were developed as guidance throughout the 20-day study period. Daily follow-up of the vancomycin treatment in trial participants is provided by trained pharmacists. This includes on-call service beyond the pharmacy opening hours and follow-up during weekends. In addition to direct communication by phone, notes and recommendations are systematically recorded in the electronic medical record (EMR) to be visible for the physicians and nurses. Feedback meetings for study coordinators, prescribers, and nursing staff are organized on a regular basis.

### Relevant concomitant care permitted or prohibited during the trial {11d}

N/A: when a participant requires extracorporeal treatment (dialysis, extracorporeal membrane oxygenation, body cooling), the trial intervention discontinues and the patient receives standard-of-care treatment. This is already been described in the “Criteria for discontinuing or modifying allocated interventions {11b}” section.

### Provisions for post-trial care {30}

The sponsor provided an insurance, even without fault, to cover its liability as the requesting party in case of harm caused to the patient by participation in the study.

### Outcomes {12}

#### Primary endpoint

The proportion of patients reaching target AUC24h/MIC of 400–600 between 48 and 72 h after start of vancomycin treatment. A MIC of 1 mg/L is assumed.

#### Secondary endpoints

1. The proportion of patients with (worsening) AKI from start of vancomycin treatment until 48 h after stopping vancomycin treatment.

AKI classes are defined using the KDIGO Clinical Practice Guideline for Acute Kidney Injury [16] (Tables 1 and 2).

Baseline serum creatinine is defined as the most recent documented serum creatinine value within 7 days before the start of the vancomycin therapy. If no value is available, baseline serum creatinine is estimated using the Modification of Diet in Renal Disease (MDRD) study equation assuming that the baseline estimated glomerular filtration rate (eGFR) is 75 ml/min/1.73 m^2^, in patients with no evidence of chronic kidney disease [16].

2) The proportion of patients reaching target AUC24h/MIC of 400–600 between 72 and 96 h after start of vancomycin treatment.
3) The proportion of time within target AUC24h/MIC of 400–600 from start until cessation of vancomycin treatment. A MIC of 1 mg/L is assumed.

**Table 1:** Definition of AKI [16]

AKI is defined as any of the following (not graded): - Increase in SCr by ≥ 0.3 mg/dl within 48 h - Increase in SCr to ≥ 1.5 times baseline, which is known or presumed to have occurred within the prior 7 days

**Table 2:** Staging of AKI [16]

Stage	Serum creatinine
1	1.5–1.9 times baseline OR ≥ 0.3 mg/dl increase
2	2.0–2.9 times baseline
3	3.0 times baseline OR Increase in serum creatinine to ≥ 4.0 mg/dl OR Initiation of renal replacement therapy

#### Tertiary endpoints

1. The number of blood samples until first target attainment during vancomycin treatment.

The target range definition:

- Interventional arm: AUC24h/MIC of 400–600 mg × h/L assuming a MIC of 1 mg/L;
- Comparator arm: target concentration range for continuous dosing regimens according to institutional guidelines.

2) The cumulative number of blood samples during vancomycin treatment.
3) The number of dose adjustments until first target attainment.

Target range definition:

- Interventional arm: AUC24h/MIC of 400–600 mg × h/L.
- Comparator arm: target concentration range for continuous dosing regimens according to institutional guidelines.

4) The cumulative vancomycin dose and AUC from start until cessation of vancomycin treatment.

### Participant timeline {13}

Participant timeline is shown in Table 3.

**Table 3:**
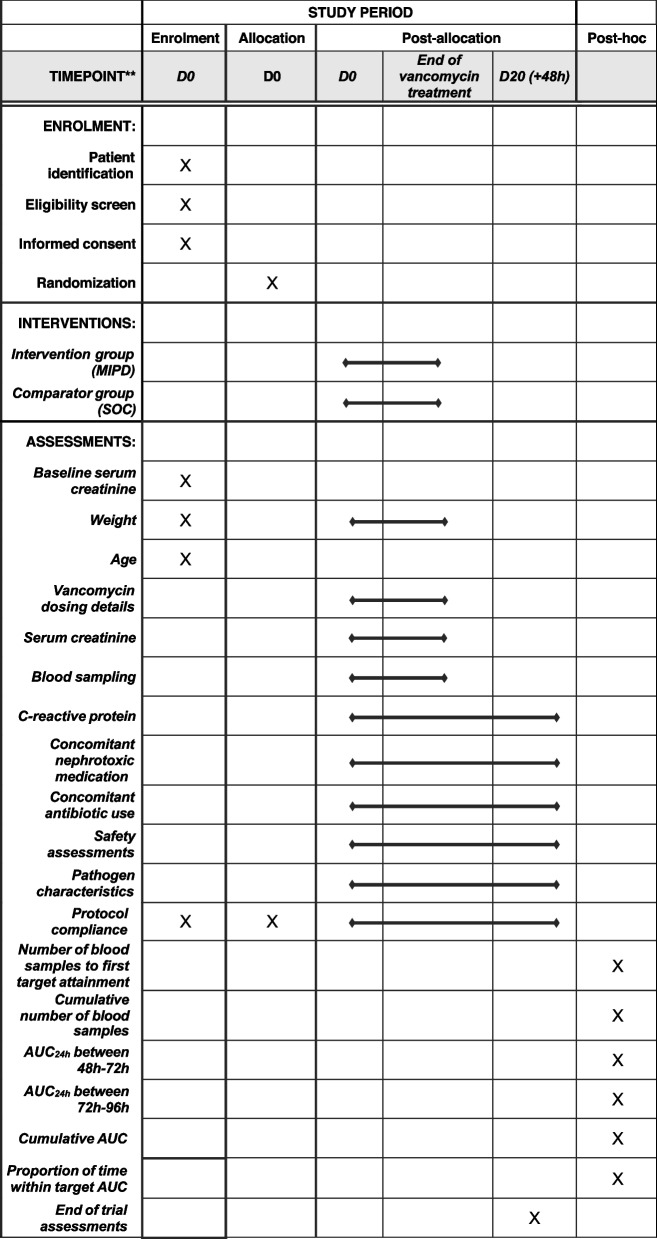
Schedule of enrolment, interventions, and trial assessments

### Sample size {14}

 The outcome on which this sample size calculation is based is the proportion of patients reaching the target AUC24h/MIC of 400–600 between 48 and 72 h after the start of vancomycin treatment. Sample size calculation for the logistic regression on this primary outcome was based on the assumed proportions of patients reaching the therapeutic target AUC24h/MIC between 48 and 72 h after the start of treatment being 50% in the standard-of-care comparator group and 75% in the AUC based vancomycin dosing intervention group.

An interim analysis will be performed after inclusion of 40 patients. An O’Brien-Fleming approach will be applied to ensure that these additional analyses do not increase the chance for a type I error. Alpha levels defined based on the O’Brien-Fleming approach are 0.21% for the interim analysis and 4.91% for the final analysis. In total, 118 participants are required to achieve a power of 80% at a significance level of 4.91%, to detect an odds ratio (OR) of 3 when the proportion in the comparator group is 50% using logistic regression. An assignment ratio of 1:1 is applied. The recruitment process is not competitive across the sites and will be terminated when the last patient is enrolled in relation to the predefined sample size.

### Recruitment {15}

#### Patient identification

Patient identification is performed by a clinical pharmacist and can occur in two ways to minimize the chance of missed patients: (a) the clinical pharmacist generates a daily query list of vancomycin medication orders of patients on the participating ward units, and (b) physicians of the participating ward are encouraged to actively inform a clinical pharmacist when the clinical decision for starting vancomycin in continuous infusion is made.

#### Patient screening

Eligibility screening through inclusion and exclusion criteria check is performed by a clinical pharmacist and study team physician. The consulted resource for inclusion and exclusion criteria check is the patient’s medical file and confirmation of eligibility by the attending physician is asked before inclusion. Informed consent is obtained by a trained and delegated physician if the patient meets the eligibility criteria.

## Assignment of interventions: allocation

### Sequence generation {16a}

Patients are randomly allocated to the interventional arm or comparator arm at inclusion. The study is a parallel-group randomized control trial with a 1:1 allocation ratio. Randomization is stratified by medical discipline. Random permuted blocks are created with a computer random number generator with variable sizes to prevent the treatment allocation from being predicted. The stratified randomization is performed using the randomization module of REDCap® based on an allocation table provided by the statistician.

### Concealment mechanism {16b}

REDCap® is a secure web-based electronic case report form (eCRF) designed to support data capture for research studies. Before a patient can be randomized, the baseline patient characteristics (date of birth, serum creatinine), start date of vancomycin treatment, and medical discipline need to be entered in REDCap®. REDCap® automatically documents the allocation and stores all the records and the randomizations. If REDCap® is not available (due to, e.g., internet problems, login problems, scheduled update procedures), a back-up procedure with envelopes stored in the investigator site file (ISF) is followed.

### Implementation {16c}

The allocation sequence is generated in SAS version 9.4 by the statistician and imported in REDCap®. Enrolment of participants is performed by a trained clinical pharmacist guided by the previously developed REDCap® randomization tool.

## Assignment of interventions: blinding

### Who will be blinded {17a}

Participants or legal representatives are blinded for the allocation to the intervention or standard-of-care arm until the end of the study.

### Procedure for unblinding if needed {17b}

N/A: no circumstances in which unblinding is permissible are determined.

## Data collection and management

### Plans for assessment and collection of outcomes {18a}

#### Vancomycin treatment

Vancomycin dosing details are recorded by the attending nurse on an electronic medication administration sheet in the EMR. If accidently one or more doses are not recorded, maximum effort will be undertaken for retrieval of the information (e.g., ask the attending nurse who administered the drug). If not retrievable, the planned dose and start and stop timing are registered. In case a patient is randomized to the standard-of-care arm, the vancomycin target range is documented in the EMR.

#### Blood sampling for vancomycin concentration measurement

Therapeutic drug monitoring of vancomycin is standard-of-care. In both the intervention and comparator group, blood samples are drawn according to the following standard-of-care principles:

- Blood sample is drawn approximately 24 h after the start of the loading dose;
- If a sub- or supratherapeutic concentration is measured, the dosing regimen is changed and a repeat sample is taken approximately 24 h after the change of dosing regimen;
- Whenever therapeutic concentrations are reached, a repeat sample is drawn every 3 to 4 days, or sooner (e.g., in case of changing renal function, suspected therapy failure, or major surgery);
- More frequent sampling to ensure efficacious treatment, to avoid toxicity or to rule out sampling errors, is performed at the discretion of the attending physician;
- More delayed or earlier sampling for practical sampling occurs at per standard-of-care and is at the discretion of the attending physician.

Vancomycin sampling times are recorded in the electronic laboratory reports. If sampling times are accidently not recorded or not reliable, maximum effort is undertaken for retrieval of the information (e.g., ask the attending nurse who performed the blood sampling). Whether the sample is additional or not is retrievable from the electronic laboratory report, i.e., if other lab parameters are performed on the same sample or not.

#### Weight

Weight is assessed as per standard-of-care and recorded in the EMR.

#### Serum creatinine

Serum creatinine concentration measurements are collected on a regular basis as per standard-of-care for patients on vancomycin therapy and will allow AKI category assessment in both study arms (see also the “Outcomes {12}” section) until 48 h after stopping of vancomycin treatment. Sampling times and concentrations are recorded in the electronic laboratory reports. Serum creatinine concentrations are compared to the baseline value before the start of vancomycin treatment to evaluate the KDIGO criteria and stages of AKI during vancomycin treatment.

#### Concomitantly administered nephrotoxic comedication

Concomitantly administered nephrotoxic comedication during vancomycin treatment (limitative list) is recorded based on prescription data from the EMR.

#### Concomitant antibiotic use

Concomitant systemic antibiotic use in the 20-day study period is recorded based on prescription data from the EMR.

#### C-reactive protein

Available C-reactive protein measurements, collected as per standard-of-care for patients on vancomycin therapy, are recorded in the electronic laboratory reports.

#### Microbiological data

Isolated pathogen(s), site of infection, and reported sensitivity are assessed as per standard-of-care and are recorded in the electronic laboratory reports.

#### Automated or semi-automated post hoc assessments

Data reported in the eCRF are used to calculate the following outcome parameters. The total number of (additional) blood samples taken to first target attainment. This endpoint will be calculated using the date of randomization and the number of (additional) vancomycin measurements between start and reported time of target attainment. The cumulative number of blood samples during therapy will be calculated by counting the number of (additional) vancomycin measurements.

#### AUC estimation

In the interventional arm, the loading dose and starting continuous infusion dose are initiated by the attending physician according to the institutional dosing regimen guidelines. For dose adjustments, the AUC24h is estimated within a maximum of 8 h after the TDM concentration is available in the laboratory report. Variables in the PK model by Colin et al (weight, age, measured serum creatinine, dosing history and TDM concentration) are entered in the software, targeting an AUC24h of 400 to 600 between 24 and 48 h after the dose calculation.

The following estimations will be performed, after the patient end of trial assessment using all available measurements per patient:

- AUC24h between 48 and 72 h (primary endpoint) and between 72 and 96 h (secondary endpoint) will be estimated for patients in the comparator arm and interventional arm;
- The cumulative AUC (tertiary endpoint) in the comparator arm and interventional arm will be estimated from start until 48 h after stopping treatment;
- The proportion of time within a target AUC24h of 400-600 (secondary endpoint) in the comparator arm and interventional arm will be estimated from start until stop treatment.

#### End of trial assessments

End of trial assessments are collected on day 20 (or day 20 plus 48 h in case of a patient still receiving vancomycin treatment on day 20) and include:

- Safety assessments;
- Whether or not the patient is (still) receiving vancomycin;
- The date and reason for end of trial including protocol completion, patient death or patient withdrawal of informed consent.

### Plans to promote participant retention and complete follow-up {18b}

The rate of loss-to-follow-up is considered to be low since vancomycin is only administered during the hospital stay. The study team consisting of investigators, clinical pharmacists, and study coordinators conduct daily rigorous patient follow-up and monitor protocol compliance. If a patient is withdrawn from the study or the study intervention is prematurely discontinued, the patient will receive standard-of-care treatment and maximum effort to continue collecting data until the end of trial is made. Protocol deviations are recorded from randomization until end of trial in a protocol deviation log in the eCRF. If applicable, a proposed plan of action for resolution will also be documented on the protocol deviation log.

### Data management {19}

#### Source data

Source documents are consulted/used and include hospital records, study worksheets, data entered in the MIPD software, and data collected during the screening visit until discharge form the hospital or until day 20.

#### Electronic case report forms (eCRF)

An electronic data capture (EDC) system, i.e., REDCap®, is used for data collection. The eCRF and the database are developed by the trial manager based on the protocol and are approved by the chief investigator (CI). Only the data required by the protocol are captured in the eCRF. If information is not known, this is clearly indicated on the eCRF. All missing and ambiguous data will be clarified.

In the comparator arm, data are transferred to the eCRF by manual data entry at the site. In the interventional arm, calculated and administered vancomycin dosing details and blood sampling details are registered in the MIPD software tool by the end-users. The trial manager will use an export file from the study data in the software that will be imported into the REDCap® database. Data is checked by trained personnel (monitor, trial manager), and any errors or inconsistencies are clarified.

#### Data handling and record keeping

REDCap® is a web-based system provided and maintained by Vanderbilt University. A license for use was granted to the Health, Innovation and Research Institute of UZ Ghent. The data are accessed through a web browser directly on the secure REDCap® server. The server is hosted within the UZ Ghent campus and meets hospital level security and back-up requirements. Privacy and data integrity between the user’s browser and the server is provided by mandatory use of Transport Layer Security (TLS) and a server certificate issued by Trans-European Research and Education Networking Association (TERENA). Login in REDCap® is password controlled and each user has a specific role that has predefined restrictions on what is allowed in REDCap®. Any activity in the software is traced and transparent via the audit trail and log files. Data validation rules are added to maximize immediate validation of the data at entry. In addition, complex edit checks are programmed by the Trial Manager to check for inconsistencies within the data. Data analysis will be performed by a statistician.

Patients who are included in the study are assigned a unique study number upon their registration in REDCap®. On all documents, patients will only be identified by their study number. The subject identification list is safeguarded at the site, and the name and any other directly identifying details will not be included in the study database. The unique study number, generated in the REDCap® randomization database, will also be used for data collection in the eCRF study database in REDCap® and to identify patients in the web-based MIPD platform. Access to the data is limited to the minimum number of individuals necessary for quality control, audit, and analysis. The data access in the MIPD platform is via a web browser directly on a secure server. The server meets General Data Protection Regulation (GDPR) security and back-up requirements. Site access for the MIPD platform is controlled with password control and each user has restricted access to the data of individual patients of the center. The clinical pharmacist is responsible for data entry in the MIPD platform. Any activity in the software is traced and transparent via the audit trail and log files. The sponsor is the data custodian.

### Confidentiality {27}

All investigators and trial staff must comply with the requirements of the Belgian and European Privacy legislation (https://www.dataprotectionauthority.be/legislation-and-standards.) on the protection of privacy in relation to the processing of personal data, with regard to the collection, storage, processing, and disclosure of personal information. A contract with the software company includes GDPR compliance requirements. Archiving will be authorized by the sponsor following submission of the end of study report. The investigator and sponsor specific essential documents will be retained for at least 25 years after the end of the trial. At that moment, it will be judged whether it is necessary to retain them for a longer period, according to applicable regulatory or other requirement(s).

### Plans for collection, laboratory evaluation, and storage of biological specimens for genetic or molecular analysis in this trial/future use {33}

Blood samples for vancomycin concentration or serum creatinine measurement are drawn as per standard of care for both intervention as comparator study groups.

## Statistical methods

### Statistical methods for primary and secondary outcomes {20a}

#### Primary outcome analysis

A logistic regression for binary data will be applied, reaching the target AUC/MIC between 48 and 72 h after starting vancomycin as categorical outcome variable and randomization group (interventional versus the standard-of-care arm) as categorical predictor of interest. The stratification factor medical discipline will be added as a covariate in the model [17]. The intervention effect will be expressed by using the OR, together with the estimated proportion per group.

#### Secondary outcome analysis

For the secondary endpoints “proportion of patients with (worsening) acute kidney injury until 48 h after stopping vancomycin treatment” and “proportion of patients reaching target AUC24h/MIC between 72 and 96 h after the start of vancomycin treatment,” the statistical methods will be similar to those described in the “Primary outcome analysis” section. We will express the intervention effect by using the OR, together with the estimated proportion per group. For the secondary endpoint “proportion of time within target range,” linear regression with randomization group and medical discipline as categorical predictor variables will be applied. We will express the intervention effect by using the mean difference.

### Interim analyses {21b}

An interim analysis for efficacy will be conducted after the inclusion and end of trial of 40 patients. We report 99.79% confidence intervals to indicate the precision of the estimates in this interim analysis. A *p*-value is reported for the comparison between arms of the primary outcome and compared to an 0.21% alpha level. This adjusted alpha level is based on the O’Brien-Fleming approach. The significance level for the interim analysis for efficacy on the primary endpoint is 0.02%. Only the CI and trial manager have access to the interim results and decide whether to continue or terminate the trial and proceed with the analyses related to the secondary and tertiary objectives.

### Methods for additional analyses (e.g., subgroup analyses) {20b}

A subgroup analysis will be performed for the stratification factor medical discipline. Statistical methods will be similar to those described in the “Primary outcome analysis” section. Medical discipline will be added as a categorical predictor variable. An interaction term between medical discipline and randomization group will be included in the model. A second subgroup analysis will be applied on the patients with confirmed infection at baseline. Statistical methods will be the same as to those described in the “Primary outcome analysis” section but applied only on the subgroup with confirmed infection.

### Methods in analysis to handle protocol non-adherence and any statistical methods to handle missing data {20c}

The primary analysis will be the intention-to-treat (ITT) analysis. All participants will be included in the analysis in the groups to which they were originally assigned, regardless of what subsequently occurred. Imputation techniques will be used for outcome data that are missing [18]. Multivariate imputation (MI) by fully conditional specification will be used to apply multiple imputation of missing data, more specifically the MI procedure in SAS with the FCS statement. The predictors used for the imputation model will include (but are not limited to): randomization group, ward unit, age of the participant, indication for vancomycin treatment, admission diagnosis, nephrotoxic medication, duration of treatment, and vancomycin dose. Primary analysis will be based on the multiple imputed data. As a sensitivity analysis, the results will be compared with the analysis on the protocol-compliant sample.

### Plans to give access to the full protocol, participant-level data, and statistical code {31c}

N/A: there are no plans for granting public access to the full protocol, dataset, and statistical code.

## Oversight and monitoring

### Composition of the coordinating center and trial steering committee {5d}

The trial steering committee (TSC) consists of the roles that are mentioned below along with their key responsibilities. The members of the TSC meet once a year to oversee the course of the study.

#### Local principal investigators (local PI)

- Checking for reportable events during the study period intervention and follow-up;
- Using medical judgment in assigning seriousness, causality, and expectedness using the Reference Safety Information approved for the trial and in consultation with the sub-investigator(s);
- Ensure that reportable events are recorded and reported in line with the requirements of the protocol;
- Checking research protocol compliance.

#### Medical principal investigator (medical PI)

- Checking for reportable events during the study period intervention and follow-up;
- Ensure that reportable events are recorded and reported in line with the requirements of the protocol;
- Checking research protocol compliance;
- Clinical oversight of the safety of patients participating in the trial, including an ongoing review of the risk/benefit;
- Using medical judgment in assigning seriousness, causality, and expectedness of SAEs/SARs where it has not been possible to obtain local medical assessment;
- Immediate review of all SUSARs;
- Review of specific SAEs and SARs in accordance with the trial risk assessment and protocol as detailed in the trial monitoring plan;
- Assigning Medical Dictionary for Regulatory Activities (MedDRA) or body system coding to all SAEs and SARs;
- Preparing of the annual progress report, including safety summary and deviations in close collaboration with the trial manager and CI;
- Preparing the clinical sections and final sign off of the Development Safety Update Report (DSUR).

#### Chief investigator (CI)

- Trial coordination in close collaboration with the trial manager;
- Responsible for training end-users on software;
- Registration of end-users, in collaboration with the trial manager;
- Immediate review of all SUSARs, in close interaction with the MPI;
- Review of specific SAEs and SARs in accordance with the trial risk assessment and protocol as detailed in the trial monitoring plan;
- Preparing of the annual progress report, including safety summary and deviations in close collaboration with the trial manager and MPI.

#### Sponsor—Ghent University Hospital

- Central data collection and verification of reportable events in the eCRF;
- Expedited reporting of SUSARs to the Federal Agency for Medicines and Health Products (FAMHP) and Ethics Committee (EC) within required timelines;
- Notifying investigators of SUSARs that occur within the trial;
- Checking for (annually) and notifying PIs of updates to the Reference Safety Information for the trial;
- Preparing standard tables and other relevant information for the DSUR in collaboration with the CI and ensuring timely submission to the FAMHP and REC;
- Submission of the annual progress reports, including safety summaries and deviations.

The trial management group (TMG) consists of the roles listed below accompanied by the CI and MPI. They meet on a frequent basis for briefing or feedback moments and to adjust day-to-day operation if necessary.

#### Trial manager

- Coordinating role during study recruitment;
- Development of the eCRF and the database based on the protocol;
- Program complex edit checks to check for inconsistencies within the data;
- Check of data entries and clarify any errors and inconsistencies;
- Conduct automatic transfer of data from the MIPD tool to the eCRF.

#### Study coordinators

- Coordinating role in obtaining informed consent from the participants;
- Patient screening according to the inclusion and exclusion criteria, in collaboration with physician and clinical pharmacist.

### Composition of the data monitoring committee, its role and reporting structure {21a}

N/A: there is no data monitoring committee because minimal risk is associated with the study. MIPD of vancomycin is already within marketing authorization.

### Adverse event reporting and harms {22}

#### Safety assessments

Following severe adverse events (SAE) occurring until 48 h after the last vancomycin, study administration should be reported in both study arms:

- KDIGO stage 3 of AKI;
- Vancomycin infusion reaction (“red man syndrome”).

Other significant safety issues (including death) at the discretion of the investigator until day 20 plus 48 h thought to be at least possibly related to the vancomycin dosing method should be reported in both study arms. These reportable safety events are assessed on a continuous basis in both study arms and are reported using an appropriate reporting form within 24 h of the research staff becoming aware of the event. Assessment of seriousness, causality, and expectedness must be made by the MPI, local PI, or an authorized physician. Events must be followed up until the event has resolved or a final outcome has been reached.

Suspected unexpected serious adverse reactions (SUSAR) being SAEs suspected to be related to vancomycin treatment or vancomycin treatment method and unexpected assigned by the MPI or local PI will be subject to expedited reporting to the FAMHP.

If any urgent safety measures are taken, the PI or CI will inform the sponsor immediately and in any event no later than 3 days from the date the measures are taken. The sponsor gives written notice to the FAMHP and the central EC of the measures taken and the circumstances giving rise to those measures. This reporting will be done in accordance with the European Medicines Agency (EMA) guidelines.

Following a reportable event, every effort should be made to ensure proper follow-up within a reasonable time after in accordance with the nature and extent of the event. Any SUSAR related to vancomycin will need to be reported to the sponsor irrespective of how long after vancomycin administration the reaction has occurred.

The CI will provide (in addition to the expedited reporting above) DSURs once a year throughout the clinical trial, or on request, to the competent authority (FAMHP in Belgium), EC, and sponsor. This DSUR will include all SAEs. The report will be submitted 1 year (+ maximum 60 days) after the “Development International Birth Date (DIBD)” and will subsequently be submitted each year until the study is declared to have ended. This DIBD is the date of the sponsor’s first overall authorization to conduct the clinical trial.

### Frequency and plans for auditing trial conduct {23}

Regular on-site and remote monitoring will be performed by the sponsor’s CTU HIRUZ according to the International Conference on Harmonization of Technical Requirements for Registration of Pharmaceuticals for Human Use (ICH) Good Clinical Practice (GCP) E6 (R2) and all applicable regulatory requirements. Prior to commencing recruitment, the monitor will meet the study team by a trial initiation visit to review trial specific procedures such as the protocol, safety reporting procedures, ICF procedures, ISF content, CI and PI’s responsibilities, eCRF completion guidelines, and study timelines. Following written standard operating procedures (SOPs), the monitors will verify on-site whether the clinical trial is conducted and data are generated, documented, and reported in compliance with the protocol, Good Clinical Practice (GCP), and the applicable regulatory requirements. Data recorded in the eCRF will be evaluated for accuracy in relation to source documents. The monitor will provide a monitoring report after each visit for the sponsor and the investigator. The frequency, extent, and nature of monitoring will be defined in more detail in a monitoring plan. Depending on the quality of the data, additional monitoring visits may be necessary according to the sponsor’s discretion.

### Plans for communicating important protocol amendments to relevant parties (e.g., trial participants, ethical committees) {25}

During the trial, all protocol amendments and revised ICF are sent to the central EC for their review. Amendments are not implemented without prior review and documented approval/favorable opinion from the EC except when necessary to eliminate an immediate hazard to trial subjects or when the changes involve only logistical or administrative aspects of the trial. After approval from the EC, important protocol modifications are directly communicated to the relevant parties. Depending on the type of modifications, communication can be by a meeting of the trial steering committee and/or trial management group, contacting trial participants, a newsletter for participating ward units, etc.

### Dissemination plans {31a}

Dissemination of the study findings will be in accordance with the EU GDPR of 14 April 2016 and the Belgian Law of 22 August 2002 on patients’ rights. All case report forms and other data (including without limitation, written, printed, graphic material, and information contained in any computer database or computer readable form) created or developed during the course of the study shall be the property of the sponsor. Open access publication, scope of the journal, and editorial facilitators to disseminate the study findings will be considered by the study team for the final selection of the targeted journal. Any funding or logistical support will be acknowledged within the journal in the appropriate section of the manuscript. Review and publication rights will be respected according to the journal policy.

## Discussion

N/A: no other issues need to be covered.

### Trial status

**Protocol version number and date**. Version 3.0, April 21, 2023. **Date of start recruitment. **November 12, 2021. **Approximate date of trial completion. **December, 2023.

## Abbreviations

AKI: Acute kidney injury
AUC: Area under the concentration-time curve
CI: Chief investigator
DIBD: Development International Birth Date
DSUR: Development Safety Update Report
EC: Ethics committee
eCRF: Electronic case report form
EDC: Electronic data capture
eGFR: Estimated glomerular filtration rate
EMA: European medicines agency
EMR: Electronic medical record
EU: European union
EudraCT: European union drug regulating authorities clinical trials database
FAMHP: Federal agency for medicines and health products
GCP: Good clinical practice
GDPR: General data protection regulation
ICF: Informed consent form
ICH: International conference on harmonization of technical requirements for registration of pharmaceuticals for human use
ICU: Intensive care unit
ISF: Investigator site file
ITT: Intention-to-treat
KDIGO: Kidney disease improving global outcomes
MDRD: Modification of diet in renal disease
MedDra: Medical dictionary for regulatory activities
MI: Multivariate imputation
MIC: Minimal inhibitory concentration
MIPD: Model-informed precision dosing
MRSA: Methicillin-resistant *Staphylococcus aureus*
OR: Odds ratio
PD: Pharmacodynamic(s)
PI: Principal investigator
PIS: Participant information sheet
PK: Pharmacokinetic(s)
QA: Quality assurance
QC: Quality control
QOL: Quality of life
RCT: Randomized control trial
RIFLE: Risk, injury, failure; loss, end-stage renal disease
SAE: Serious adverse event
SAR: Serious adverse reaction
SmPC: Summary of product characteristics
SOC: Standard of care
SOP: Standard operating procedure
SUSAR: Suspected unexpected serious adverse reaction
TDM: Therapeutic drug monitoring
TERENA: Trans-european research and education networking association
TLS: Transport layer security
TMG: Trial management group
TSC: Trial steering committee

## References

[1] Liu C, Bayer A, Cosgrove SE, Daum RS, Fridkin SK, Gorwitz RJ, et al. Clinical practice guidelines by the Infectious Diseases Society of America for the treatment of methicillin-resistant Staphylococcus aureus infections in adults and children. Clin Infect Dis. 2011;52(3):e18–55. 21208910 10.1093/cid/ciq146

[2] Kabbara WK, El-Khoury G, Chamas NR. Prospective evaluation of vancomycin therapeutic usage and trough levels monitoring. J Infect Dev Ctries. 2018;12(11):978–84. 32012127 10.3855/jidc.9800

[3] Rybak MJ, Le J, Lodise TP, Levine DP, Bradley JS, Liu C, et al. Therapeutic monitoring of vancomycin for serious methicillin-resistant Staphylococcus aureus infections: a revised consensus guideline and review by the American Society of Health-System Pharmacists, the Infectious Diseases Society of America, the Pediat. Am J Health Syst Pharm. 2020;77(11):835–64. 32191793 10.1093/ajhp/zxaa036

[4] Aljefri DM, Avedissian SN, Rhodes NJ, Postelnick MJ, Nguyen K, Scheetz MH. Vancomycin area under the curve and acute kidney injury: a meta-analysis. Clin Infect Dis. 2019;69(11):1881–7. 30715208 10.1093/cid/ciz051 PMC6853683

[5] Tängdén T, Ramos Martín V, Felton TW, Nielsen EI, Marchand S, Brüggemann RJ, et al. The role of infection models and PK/PD modelling for optimising care of critically ill patients with severe infections. Intensive Care Med. 2017;43(7):1021–32. 28409203 10.1007/s00134-017-4780-6

[6] Darwich AS, Ogungbenro K, Vinks AA, Powell JR, Reny JL, Marsousi N, et al. Why has model- informed precision dosing not yet become common clinical reality? Lessons from the past and a roadmap for the future. Clin Pharmacol Ther. 2017;101(5):646–56. 28182269 10.1002/cpt.659

[7] Norris RL, Martin JH, Thompson E, Ray JE, Fullinfaw RO, Joyce D, et al. Current status of therapeutic drug monitoring in Australia and New Zealand: a need for improved assay evaluation, best practice guidelines, and professional development. Ther Drug Monit. 2010;32(5):615–23. 20683393 10.1097/FTD.0b013e3181ea3e8a

[8] Neely MN, Kato L, Youn G, Kraler L, Bayard D, van Guilder M, et al. Prospective trial on the use of trough concentration versus area under the curve to determine therapeutic vancomycin dosing. Antimicrob Agents Chemother. 2018;62(2). 10.1128/AAC.02042-17 PMC5786789 29203493

[9] Nederlandse Vereniging van Ziekenhuisapothekers. TDM Monografie.org - Vancomycine [Internet]. [cited 2021 Apr 14]. Available from: https://tdm-monografie.org/monografie/Vancomycine.

[10] Antibiotic Expert Group of Therapeutic Guidelines Limited. Therapeutic guidelines: antibiotic. Version 14 [Internet]. 2023. Available from: http://www.tg.org.au/index.php?sectionid=41.

[11] Ampe E, Delaere B, Hecq JD, Tulkens PM, Glupczynski Y. Implementation of a protocol for administration of vancomycin by continuous infusion: pharmacokinetic, pharmacodynamic and toxicological aspects. Int J Antimicrob Agents. 2013;41(5):439–46. 23523733 10.1016/j.ijantimicag.2013.01.009

[12] Saugel B, Gramm C, Wagner JY, Messer M, Lahmer T, Meidert AS, et al. Evaluation of a dosing regimen for continuous vancomycin infusion in critically ill patients: an observational study in intensive care unit patients. J Crit Care. 2014;29(3):351–5 https://www.sciencedirect.com/science/article/pii/S0883944113004759. 24456810 10.1016/j.jcrc.2013.12.007

[13] Insight RX Inc. Insight Rx Nova (Version 1.36.6).[Software]. 2021. Available from: insight-rx.com.

[14] Colin PJ, Allegaert K, Thomson AH, Touw DJ, Dolton M, de Hoog M, et al. Vancomycin pharmacokinetics throughout life: results from a pooled population analysis and evaluation of current dosing recommendations. Clin Pharmacokinet. 2019;58(6):767–80. 30656565 10.1007/s40262-018-0727-5

[15] Heus A, Uster DW, Grootaert V, Vermeulen N, Somers A, Huis in ‘t Veld D, et al. Model-informed precision dosing of vancomycin via continuous infusion: a clinical fit-for-purpose evaluation of published PK models. Int J Antimicrob Agents. 2022:106579. https://www.sciencedirect.com/science/article/pii/S0924857922000796. 10.1016/j.ijantimicag.2022.106579 35341931

[16] Khwaja A. KDIGO clinical practice guidelines for acute kidney injury. Nephron Clin Pract. 2012;120(4):c179–84. 22890468 10.1159/000339789

[17] European Medicines Agency. ICH E9 statistical principles for clinical trials (CPMP/ICH/363/96) [Internet]. [cited 2021 Apr 15]. Available from: https://www.ema.europa.eu/en/ich-e9-statistical-principles-clinical-trials.

[18] Moher D, Hopewell S, Schulz KF, Montori V, Gøtzsche PC, Devereaux PJ, et al. CONSORT 2010 explanation and elaboration: updated guidelines for reporting parallel group randomised trials. BMJ. 2010;340:c869. 20332511 10.1136/bmj.c869 PMC2844943

